# Multifactor Analyses of Frontal Cortex Lipids in the APP/PS1 Model of Familial Alzheimer’s Disease Reveal Anomalies in Responses to Dietary n-3 PUFA and Estrogenic Treatments

**DOI:** 10.3390/genes15060810

**Published:** 2024-06-19

**Authors:** Mario Díaz

**Affiliations:** 1 Membrane Physiology and Biophysics, Department of Physics, School of Sciences, University of La Laguna, 38206 Tenerife, Spain; madiaz@ull.es or madiaz@ull.edu.es; 2 Instituto Universitario de Neurociencias (IUNE), University of La Laguna, 38206 Tenerife, Spain

**Keywords:** neurolipids, Alzheimer’s disease, APP/PS1 mice, LCPUFAs (long-chain polyunsaturated fatty acids), plasmalogens, estrogens, phospholipid remodeling, membrane viscosity

## Abstract

Brain lipid homeostasis is an absolute requirement for proper functionality of nerve cells and neurological performance. Current evidence demonstrates that lipid alterations are linked to neurodegenerative diseases, especially Alzheimer’s disease (AD). The complexity of the brain lipidome and its metabolic regulation has hampered the identification of critical processes associated with the onset and progression of AD. While most experimental studies have focused on the effects of known factors on the development of pathological hallmarks in AD, e.g., amyloid deposition, tau protein and neurofibrillary tangles, neuroinflammation, etc., studies addressing the causative effects of lipid alterations remain largely unexplored. In the present study, we have used a multifactor approach combining diets containing different amounts of polyunsaturated fatty acids (PUFAs), estrogen availabilities, and genetic backgrounds, i.e., wild type (WT) and APP/PS1 (FAD), to analyze the lipid phenotype of the frontal cortex in middle-aged female mice. First, we observed that severe n-3 PUFA deficiency impacts the brain n-3 long-chain PUFA (LCPUFA) composition, yet it was notably mitigated by hepatic de novo synthesis. n-6 LCPUFAs, ether-linked fatty acids, and saturates were also changed by the dietary condition, but the extent of changes was dependent on the genetic background and hormonal condition. Likewise, brain cortex phospholipids were mostly modified by the genotype (FAD>WT) with nuanced effects from dietary treatment. Cholesterol (but not sterol esters) was modified by the genotype (WT>FAD) and dietary condition (higher in DHA-free conditions, especially in WT mice). However, the effects of estrogen treatment were mostly observed in relation to phospholipid remodeling in a genotype-dependent manner. Analyses of lipid-derived variables indicate that nerve cell membrane biophysics were significantly affected by the three factors, with lower membrane microviscosity (higher fluidity) values obtained for FAD animals. In conclusion, our multifactor analyses revealed that the genotype, diet, and estrogen status modulate the lipid phenotype of the frontal cortex, both as independent factors and through their interactions. Altogether, the outcomes point to potential strategies based on dietary and hormonal interventions aimed at stabilizing the brain cortex lipid composition in Alzheimer’s disease neuropathology.

## 1. Introduction

Alzheimer’s disease (AD) is the most prevalent form of dementia in developed countries. Despite intensive research in recent decades, sporadic AD, the most common form of this neuropathology, has no cure due to its complex idiopathic etiology. Though aging represents the main risk factor for the development of AD, compelling epidemiological and experimental evidence points to genetic predisposition, type of diet, onset of menopause, metabolic traits, and environmental factors, amongst others, as factors involved in the risk of developing AD.

Evidence accumulated over the last three decades has underscored that brain lipids are critical players in the pathogenesis of AD. Alterations in different brain lipids, including cholesterol, sphingolipids, and long-chain polyunsaturated fatty acids (LCPUFAs), have been observed in *postmortem* human brains even at the earliest stages of AD [1,2,3,4]. In this sense, DHA (docosahexaenoic acid, C22:6 n-3), the main n-3 LCPUFA in nerve cell phospholipids (~20% of total fatty acids), appears to be consistently reduced in different brain areas of AD patients [4,5,6]. Furthermore, epidemiological and experimental studies have highlighted the beneficial influence of DHA on the preservation of synaptic function and memory and cognitive performance in animal models of AD [6,7,8,9,10,11]. Indeed, it is now accepted that its deficiency increases the risk of cognitive impairment and developing AD [6,8,12,13].

Beyond a reproductive hormone, estradiol is also a brain-derived neurotrophic factor that modulates estrogen receptor (ER)-mediated signaling mechanisms that protect the brain from neurodegenerative diseases in both males and females [14,15,16]. Moreover, though still controversial, epidemiological studies and clinical studies suggest that estrogen treatment may prevent age-related cognitive decline and reduce the risk of dementia and AD [17,18]. Women possess greater lifetime risk of dementia compared to men due to longer life expectancy and make up approximately 65–70% of all cases of AD [19,20]. The increased prevalence and risk of AD among women have also been attributed to sudden decreases in estrogen levels post-menopause. Hitherto, the contribution of estrogens on differential lipid distribution and metabolism as well as their protective role in cognitive decline are not fully understood.

Although the neuroprotective effects of these factors have been extensively studied independently, the potential interactions between genetic, hormonal, and dietary factors on the lipid distribution in the brain cortex and its liver correlates have never been analyzed in depth in the literature. In the current study, we have used a multifactor approach endeavoring to analyze the combinatory effects of different hormonal and dietary n-3 PUFA conditions on the lipid distribution and n-3-LCPUFA-biosynthesis-related gene expression in the brain cortex of wild-type and APPswe/PS1ΔE9 transgenic mice, an established model of familial AD (FAD). The brain cortex of this transgenic model has been demonstrated to develop age-related changes linked to AD pathology, including the production of soluble amyloid-β (1-42 and 1-40) and plaque formation surrounded by dystrophic neurites, which are accompanied by oxidative damage, hypertrophic astrocytes and microglia, and malfunctioning of subcellular degradation pathways [21,22]. In addition, cognitive deficits such as impaired memory and learning performances are evident in this FAD model from the age of 6 months [21,23]. In recent years, lipidomic studies have uncovered anomalous lipid profiles of both brain cortex homogenates and nerve cell membrane microdomains occurring from the age of 6 months in this FAD model [24,25]. Nevertheless, most of these studies have been performed in male mice fed standard conditions, which disregards the influence of essential dietary components and the potential modulatory role of ovarian hormones in the brain lipidome. In this context, we have used multivariate techniques to assess the contribution of dietary n-3 LCPUFAs, circulating estrogens, and the presence of a FAD genotype, as well as their interactions, in setting the lipid signature of the female frontal cortex. The results underscore the enormous impact of diet on cortical LCPUFAs, as well as the critical influence of genotype and the presence of estrogens in fatty acids and phospholipid homeostasis. More importantly, our results point to hormonal and dietary interventions as specific nutraceutical strategies for Alzheimer’s disease, particularly during early stages of the disease.

## 2. Materials and Methods

*Animals and treatments*. AβPPswe/PS1ΔE9 transgenic mice and wild-type (WT) littermates were bred and maintained under standard housing conditions with a 12 h dark–light cycle at the Animal Facilities Service of the University of La Laguna (Spain). After weaning, genomic DNA was isolated from tails and genotyped by PCR using the conditions recommended by Jackson Laboratory (Maine, USA). Females carrying the APP/PS1 genotype and their WT littermates were housed separately and randomly assigned to the different groups in the present study.

Eight experimental groups were established according to their genotype (WT/FAD), DHA supplementation (DHA-Free or DHA-Suppl), and the presence of estradiol (17β-estradiol or vehicle as a placebo) following a 2 × 2 × 2 factorial design. The experimental groups were defined as follows: APF: APP/PS1-Placebo-DHA-Free; APS: APP/PS1-Placebo-DHA-Suppl; AEF: APP/PS1-Estradiol-DHA-Free; AES: APP/PS1-Estradiol-DHA-Suppl; WPF: Wild-type-Placebo-DHA-Free; WPS: Wild-type-Placebo-DHA-Suppl; WEF: Wild-type-Estradiol-DHA-Free; WES: Wild-type-Estradiol-DHA-Suppl.

**Scheme 1:**
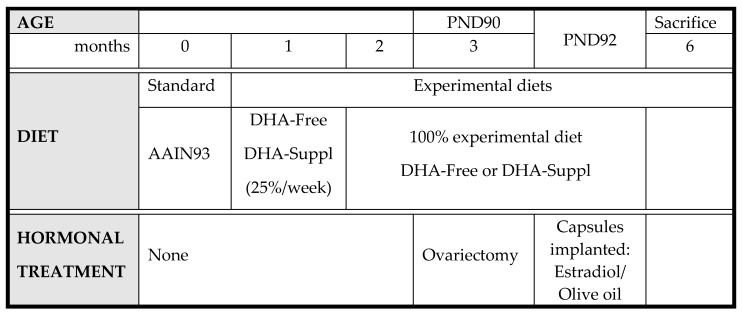
Experimental scheme for feeding WT and APP/PS1 mice.

Accordingly, WT and APP/PS1 mice were submitted to the following experimental scheme (Scheme 1):

DHA-free and -supplemented diets were prepared by Harlan Laboratories (Barcelona, Spain), and their compositions are shown in Supplementary Materials Table S1. One month after weaning, diets were gradually replaced with a DHA-free or DHA-supplemented diet at a rate of 25% per week. At postnatal day 90 (PND90), animals were ovariectomized, and 48 h later (PND92), they were implanted subcutaneously with synthetic capsules (Innovative Research of America, Sarasota, FL, USA) containing either estradiol (0.05 mg estradiol acetate/pellet) or a vehicle (olive oil). Six months after birth, mice were sacrificed by cervical dislocation. Blood samples were immediately obtained from the abdominal cava and centrifuged at 3000 rpm for 20 min at 4 °C, and the plasma fraction was collected and stored, frozen at −20 °C until assay. Brain frontal cortexes were dissected and frozen in liquid nitrogen until analysis. All experimental manipulations were performed following the procedures authorized by the Ethics Committee for the manipulation of laboratory animals at the University of La Laguna (Spain) following the guidelines of the European Community Council (Directive 86/609/EEC).

*Lipid analyses.* Lipid analyses were performed as described previously [24,26,27]. Briefly, total lipids from the frontal cortex were extracted with chloroform/methanol (2:1 *v*/*v*) containing 0.01% of butylated hydroxytoluene as an antioxidant. Lipid classes were separated using one-dimensional double-development HPTLC (high-performance thin layer chromatography) with hexane/diethyl ether/acetic acid (22.5:2.5:0.25 volume basis) as a solvent system for neutral lipid classes and methyl acetate/isopropanol/chloroform/methanol/0.25% KCl (5:5:5:2:1.8 volume basis) as a solvent system for polar lipid classes. Lipid classes were quantified using scanning densitometry after charring plates with 3% (*w*/*v*) aqueous cupric acetate containing 8% (*v*/*v*) phosphoric acid.

The fatty acid composition was determined from total lipids upon acid-catalyzed transmethylation for 16 h at 50 °C, using 1 mL of toluene and 2 mL of 1% sulfuric acid (*v*/*v*) in methanol. The resultant fatty acid methyl esters (FAMEs) and dimethylacetals (DMAs) were purified using thin layer chromatography. FAMEs were separated and determined in a TRACE GC Ultra (THERMO) gas chromatograph equipped with a flame ionization detector. Individual FAMEs and DMAs were identified by reference to a multi-standard mixture (Supelco PARK, Bellefonte, PA, USA) and confirmed using a DSQ II mass spectrometer (Thermo Fisher Scientific, Waltham, MA, USA).

*Determination of mRNA levels by Real-Time RT-PCR.* Relative *mRNA* expression levels were performed as described previously (Diaz et al., 2016). Briefly, total RNA was purified from the frontal cortex and liver and preserved in RNAlater^TM^ (Invitrogen, Waltham, MA, USA) using the RNeasy^®^ Lipid Tissue Kit Mini Kit (Qiagen, Hilden, Germany), followed by DNase I digestion, phenol:chloroform extraction and ethanol precipitation. cDNA samples were obtained with the Transcriptor First Strand Synthesis Kit (Roche, Basel, Switzerland). Amplification primers were targeted to different exons or exon/exon boundaries, as described in detail in Diaz et al. (2016). An RT-PCR was performed using SYBR Green detection on a LightCycler 480 platform (Roche, Basel, Switzerland).

*Statistics*. Lipid variables were initially assessed using one-way analysis of variance (one-way ANOVA) followed by Tukey’s or Games–Howell *post hoc* tests, where appropriate. Kruskal–Wallis and Mann–Whitney U tests were used in cases where normality or homoscedasticity was not achieved. In order to determine the main effects between different factors, and to assess the existence and degree of their interaction, data were submitted to two-way analysis of variance (two-way ANOVA). Lipid classes and the main fatty acids were additionally submitted to multivariate analysis by means of principal component analysis (PCA), in order to obtain the extraction coefficient matrixes of lipid components and their contributions to overall variance and weights in group segregation. Factor scores were further analyzed using two-way ANOVA to evaluate the main effects of diet, hormonal status, and genotype, as well as their interactions, in the lipid signatures of the different groups. Linear regression, Pearson’s correlation and partial correlation analyses were performed to assess linear relationships between different lipid variables, and to explore for the effects of individual factors controlling for bivariate relationships. Additional covariance analyses (ANCOVA) were performed to seek statistical differences in regression models and to look for the involvement of covariates in the analyses of lipid groups’ contents. Effect sizes were determined using Cohen’s *d* for two group comparisons, Cohen’s *f*^2^ for linear regressions and partial eta squared (η^2^) for multifactor analyses [28,29]. The SPSS 22.0 (IBM, New York, NY, USA) software package was used throughout the study.

## 3. Results and Discussion

The fatty acid composition of the frontal cortex is differentially affected by the dietary condition, the presence of FAD genotype and circulating estrogens.

**Figure 1:**
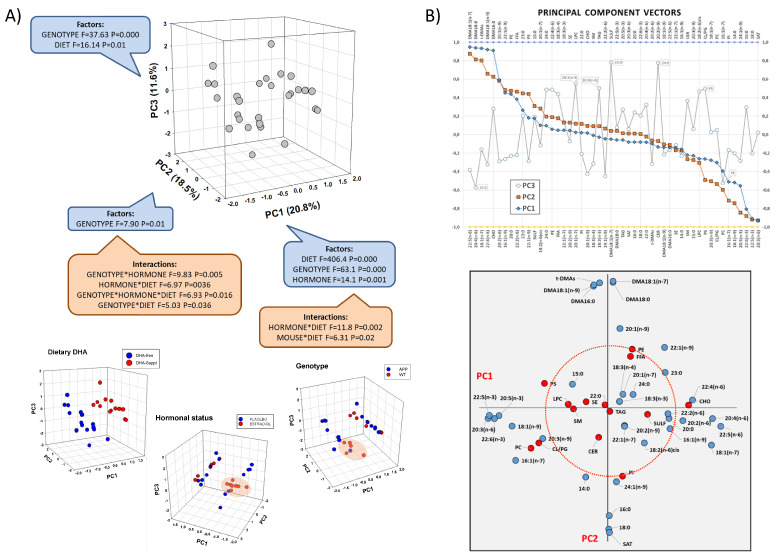
*Multivariate analyses of frontal cortex lipid composition using principal component analysis (PCA).* (**A**) Three-dimensional scatterplots for factor loadings in principal components 1, 2 and 3 (PC1, PC2, and PC3). The fraction of total variance explained by each principal component is indicated between parentheses. Results from two-way ANOVA for main factors and interactions are shown for each principal component. Lower panel: 3D scatter plots for each individual factor in the study indicating factor categories (dietary condition: DHA-Free and DHA-Suppl; hormonal treatment: placebo and estradiol; genotype: WT and FAD). (**B**) Upper panel: extraction coefficients of lipid variables in each principal component vector in the rotated component matrix. Lower panel: 2D scatterplot for variable loadings in PC1 and PC2. Red circumscribed circle in the by-plot corresponds to the value of 0.5 in each axis and includes most lipid classes. CER: cerebrosides, CHO: cholesterol, FFA: free fatty acids, LPC: lysophosphatidylcholine, PC: phosphatidylcholine, PE: phosphatidylethanolamine, PG: phosphatidylglycerol, PI: phosphatidylinositol, PS: phosphatidylserine, SAT: saturates, SE: sterol esters, SM: sphingomyelin, SULF: sulfatides, TAG: triacylglycerols, t-DMAs: total dimethylacetals.

We initially used a multivariate approach to assess the potential effects of experimental factors in the frontal cortex lipidome. Principal component analysis (PCA) of frontal cortex lipid species revealed differential contributions to the overall variance of fatty acids and lipid classes, which could be satisfactorily resolved in a three-principal-component space (Figure 1A). The three principal components (PC1, PC2 and PC3) accumulated more than 51% of total variance, each with significant loadings. Two-way ANOVA showed that the dietary condition was the main factor determining group differences and was mostly assigned to PC1 (F_1,40_ = 406.4, *p* = 0.000), while genotype was mostly determinant of PC2 (F_1,40_ = 7.9, *p* = 0.01), and both factors converged in PC3 (Figure 1A). The contribution of the hormonal status was significant in PC1 (F_1,40_ = 14.1, *p* = 0.001) but was much more relevant in terms of interactions with both, diet in PC1 (F_1,40_ = 11.8, *p* = 0.002) and genotype in PC2 (F_1,40_ = 9.83, *p* = 0.005).

Analysis of component vectors, as shown in Figure 1B, indicated that lipid variables’ highest extraction coefficients in PC1 and PC2 were fatty acids. Indeed, long-chain n-6 PUFAs (docosapentaenoic acid-22:5n-6-, arachidonic acid-20:4n-6-, adrenic acid, -22:4n-6-) were positively related in PC1, while long-chain n-3 PUFAs (docosapentaenoic acid-22:5n-3-, docosahexaenoic acid-22:6n-3-, eicosapentaenoic acid-20:5n-3-) and monoenoic 18:1n-9 (oleic acid) were negatively associated in PC1. In the case of PC2, 16- and 18-carbon dimethylacetals (DMAs) (derived from plasmalogens) were positively related, and negatively to the most abundant saturates palmitic and stearic acids (16:0 and 18:0, respectively). Of note, fatty acid variables with the highest coefficients in PC1 were poorly represented in PC2 and vice versa (Figure 1B, lower panel), indicating differential modulation by diet (the main factor for PC1) and genotype (the main factor for PC2). Consistently, the perfect segregation of DHA-Free and DHA-Supplemented groups evidenced in the plots of factor loadings shows a degree of intersection with the genotype and hormonal status superimposed on the plots of dietary segregation (Figure 1A, 3D plots in lower panel).

**Figure 2:**
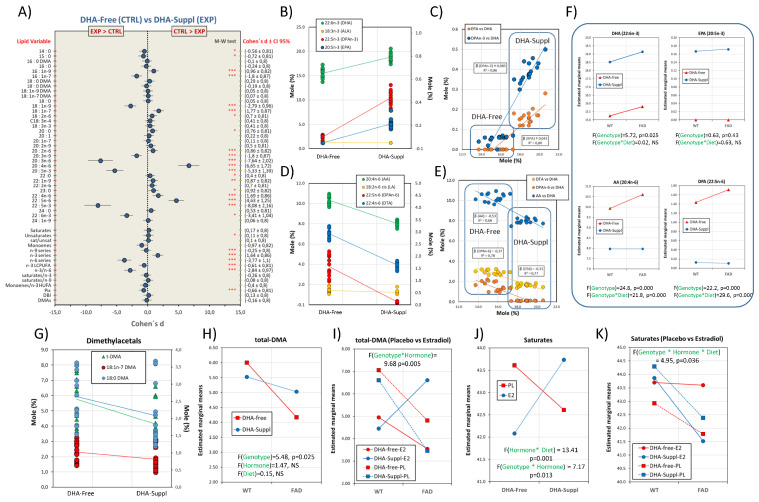
Frontal cortex fatty acid profiles are differentially affected by the dietary condition, the presence of FAD genotype and circulating estrogens: multifactor analyses. (**A**) Forest plot for effect sizes (Cohen’s d) in fatty acids and related indexes between DHA-Free and DHA-Suppl diets. Cohen’s d values are indicated along with their 95% confidence intervals. Lipid variables were also submitted to Mann–Whitney U tests. *, **, and *** indicate *p*-values below 0.05, 0.01, and 0.005, respectively. (**B**) Plots for frontal cortex contents of DHA, ALA, DPAn-3, and EPA in DHA-Free and DHA-Suppl diets. (**C**) Regression analyses for EPA and DPAn-3 as a function of DHA contents. Standardized regression coefficients (β) for each regression line are indicated along with the corresponding determination coefficient (R^2^). (**D**) Plots for frontal cortex contents of AA, LA, DPAn-6, and DTA in DHA-Free and DHA-Suppl diets. (**E**) Regression analyses for DTA, DPAn-6, and AA as a function of DHA contents. Standardized regression coefficients (β) and determination coefficient (R^2^) for each regression line. (**F**) Estimated marginal means for DHA, EPA, AA, and DPAn-6 as a function of the mouse genotype (WT and FAD). F- and *p*-values for genotype as the main factor, as well as for the interaction with the diet factor (Genotype*Diet), are shown at the bottom of each plot. (**G**) Plots for frontal cortex contents of different dimethylacetals, and total (t-DMAs) in DHA-Free and DHA-Suppl diets. (**H**) Estimated marginal means for total dimethylacetals (total-DMAs) as a function of the mouse genotype. F- and *p*-values for genotype, hormonal treatment and diet as main factors are indicated at the bottom of the graph. (**I**) Estimated marginal means for total-DMAs as a function of the mouse genotype, hormonal treatment, and dietary intervention. (**J**) Estimated marginal means for total saturates as a function of diet and hormonal treatment. F- and *p*-values for hormonal interactions with genotype (Hormone*Genotype) or diet (Hormone*Diet) are indicated at the bottom of the graph. (**K**) Estimated marginal means for total saturates as a function of genotype, hormonal treatment, and dietary condition.

The highest representation of n-3 and n-6 LCPUFAs in PC1 is a consequence of the effects of DHA supplementation in the frontal cortex fatty acid distribution. Besides LCPUFA contents, the levels of non-LCUFA fatty acids and lipid classes were also affected by the dietary supplementation, with different effect sizes, as illustrated in the forest plot shown in Figure 2A. Perhaps the most striking observation is the presence of high amounts of DHA in the frontal cortex in both dietary groups (>15% total fatty acids), despite very different provisions of the n-3 precursor α-linolenic acid (ALA, 18:3n-3) and free DHA between diets (Supplementary Materials Table S1). As the ability of brain cells to synthetize LCPUFAs from C18 precursors is very limited [30,31], the high levels of brain DHA contents in the DHA-Free group must be entirely attributable to two facts: (1) the efficient liver capacity for de novo DHA biosynthesis and (2) the high-capacity transport system transferring LCPUFAs into the brain through the blood–brain barrier [32]. Indeed, the ability of the liver–brain system to produce and transport high amounts of DHA to the brain has been consistently demonstrated and supported by a number of studies in response to a variety of dietary conditions, including severe n-3 PUFA deficiency (reviewed in [33,34,35,36]).

Even so, our data show that DHA levels were significantly lower in the DHA-Free group than in the DHA-Suppl counterparts (Figure 2B). Compared with the adequate diet (DHA-Suppl) group, the deficient diet (DHA-Free) group exhibited a reduction of 18% (for pooled marginal means) in brain DHA with a strong effect size (Cohen’s *d* = −3.41). Such a reduction is a consequence of the severe restriction of n-3 precursors in the DHA-Free diet, which is the limiting factor in the liver’s capacity to synthesize DHA. In agreement, in rodent models, a 0.9% ALA has been suggested as the threshold for homeostatic DHA levels in the brain [37,38], which is above the level used in the DHA-Free diet. These observations are physiologically relevant since brain n-3 deficiency affects nerve cell functionalities, and they have been related to deficits in cognitive and memory performance (reviewed in [10,39]) and also to psychiatric and neurodegenerative conditions [40,41].

The brain cortex contains negligible levels of the only precursor supplied in the diet (ALA), but significant levels of DHA biosynthesis intermediate 22:5n-3 (DPAn-3, docosapentaenoic acid) and 20:5n-3 (EPA, eicosapentaenoic acid), especially in the DHA-supplemented diet (Figure 2B). These observations strongly indicate two main aspects of brain PUFA metabolism: (1) most DHA biosynthesis occurs at a hepatic level and almost no ALA reaches the brain, and (2) there exists a degree of DHA retroconversion to EPA within the brain cortex in conditions of excess DHA (i.e., DHA-Suppl diet). Indeed, there are significant positive relationships between DHA levels and brain contents of EPA and DPAn-3 (Figure 2C), the slopes being higher for DPAn-3 than for EPA.

Further, detailed analyses of regression plots indicate that the significant association between DHA and EPA or DPAn-3 is limited to the DHA-Suppl group (Figure 2C), suggesting that despite significant levels of DHA in the DHA-Free groups, DHA retroconversion occurs locally at a rate of 4.1% and only above a certain threshold, estimated at about 17% total fatty acids in Figure 2B. In agreement, DHA retroconversion to EPA has been reported in brain cells as a result of excess DHA, yet there is agreement that its contribution to neural membranes is generally low [42,43]. It may be concluded that under conditions of excess DHA, conversion of n-3 PUFAs to DHA is halted or reduced to a minimum, whereby ALA is stored in cellular depots. In agreement, Igarashi and coworkers have demonstrated that the brain’s ability to synthesize DHA from ALA is very low and not altered by n-3 PUFA deprivation. Because the liver’s reported ability is much higher and can be upregulated by the deficient diet, DHA converted by the liver from circulating ALA is the source of the brain’s DHA when DHA is not included in the diet [35,44]. This may also explain genetic studies showing poor regulation of brain genes involved in LCPUFA biosynthesis in the rat cortex, even under deficient conditions, which is in contrast with the exquisite hepatic sensitivity to ALA/DHA dietary restriction.

On the other hand, levels of n-6 LCPUFAs were severely altered by dietary treatment despite no differences in the dietary supply of n-6 precursor linoleic acid (18:2n-6, LA), indicating that the level of DHA modulates the brain’s n-6 LCPUFA content (Figure 2D). In fact, we observed a negative impact of dietary DHA on the levels of arachidonic acid, as well as on the contents of 22C polyunsaturated intermediates (DPAn-6 and DTA) (Figure 2E). Such reciprocal modulation is illustrated in the negative relationship between DHA and AA as well as DPAn-6 and DTA (Figure 2E), which displayed similar slopes. These observations suggest a DHA-induced reduction in the biosynthetic pathway for AA formation.

Although the main factor responsible for LCPUFA contents in the frontal cortex is the dietary condition, PC1 was also linked to the genotype (Figure 1A). Two-way ANOVA showed that in the FAD genotype, the accumulation of 20:4n-6 is favored in the DHA-free diet (F_1,40_ = 24.84, *p* = 0.000), as is the formation of their biosynthetic intermediates, while the n-3 LCPUFAs remained unaffected by the genotype (Figure 2F). Indeed, a significant interaction between the genotype and diet was observed for AA, DPAn-6, and DTA. Similar results were obtained using linoleic acid (LA) as a covariate in the generalized linear model (ANCOVA), indicating that n-6 LCPUFA changes in the brain cortex are independent of LA elongation and desaturation. Thus, unlike brain cortex n-3 LCPUFA contents, which are relatively independent of the genotype, n-6 LCPUFA contents are modified by the diet in a genotype-dependent manner, their values being larger in FAD animals receiving a DHA-free diet (Figure 2F lower panels). These observations are relevant from a pathological point of view since a high n-6/n-3 ratio (as found in Western diets) is associated with a higher incidence of brain deficits and neuropathological states [45,46,47]. Additionally, AA is a precursor for a series of eicosanoids (i.e., series 2 prostanoids such prostaglandin E2, prostacyclin I2, and thromboxane A2 as well as series 5 leukotrienes such LTB4, LTC4, and LTE4), which behave as mediators of inflammation [32,48,49], indicating that the high n-6/n-3 proportion favors neuroinflammation. In addition, the presence of DPAn-6, which, according to our data, is exclusively associated with DHA-free diets (and negatively related to DHA), has been demonstrated to contribute to behavioral and functional abnormalities associated to dietary n-3 PUFA deprivation in rodents [50]. In agreement, increased contents of n-6-containing phospholipids and the generation of primary n-6 oxidation products have been demonstrated in the brains of rats receiving n-3-deficient diets [51]. Hence, according to our results, the FAD genotype would favor a pro-inflammatory phenotype in conditions where the DHA supply to the brain is limited to the hepatic biosynthesis from 18C PUFA precursors. In agreement, the augmented presence of inflammatory markers in the cortical parenchyma, often associated with microglial activation and astrocyte hypertrophy, has been consistently reported in different transgenic models of FAD, including in APP/PS1 transgenic mice [22,52,53].

One of the main differences between plasma and frontal cortex lipids is the presence of significant amounts of plasmalogens, reflected in the levels of dymethylacetals 18:0 DMAs, 16:0 DMAs, 18:1n-9 DMAs and 18:1n-7 DMAs (as well as total DMAs). These fatty acids were positively related to PC2 (Figure 1B, lower plot) and exhibited a significant influence of genotype as well as a degree of interaction between genotype and hormonal factors (see above and Figure 1). Detailed analyses of plasmalogens revealed a negative effect of FAD genotype consisting in a reduction in both saturated and monounsaturated dimethylacetals (Figure 2G). Two-way ANOVA showed that such a moderate-to-strong size effect (η^2^ = 0.186) reduction was significantly linked to FAD genotype independent of the dietary condition (Figure 2H). These results are particularly relevant since plasmalogens (specially PE plasmalogens) have been consistently found to be decreased in AD animal models and in human brains at advanced stages of AD, in correlation with disease severity [5,25,39,54,55]), as well as in the grey matter of young and old dementia patients [56]. Indeed, as plasmalogens need intact peroxisomes for their biosynthesis, their reduction in the frontal cortex is strongly indicative of peroxisomal dysfunction in AD, as has been suggested elsewhere [39].

Interestingly, the lower t-DMA levels in the cortex of FAD animals receiving the DHA-free diet compared to WT counterparts was reversed by the incorporation of 17β-estradiol. Such strong genotype–hormonal status interaction (η^2^ = 0.287) was clearly unmasked in the analyses of marginal means shown in Figure 2I, where the incorporation of estradiol counteracts the negative effect of the genotype on total DMAs.

Saturated fatty acids were also highly associated with PC2 (Figure 1B). The two main saturates, 16:0 (palmitic acid) and 18:0 (stearic acid), account for about 40% total fatty acids in the brain cortex, representing a main structural constituent of nerve cell membranes, and were negatively related to PC2 with high extraction coefficients. These fatty acids did not exhibit significant differences in their overall contents between DHA-Free and DHA-Suppl groups (Figure 2A) but showed slight differences depending on the genotype and hormonal treatment. Indeed, very significant and strong interactions can be demonstrated for hormonal treatment and genotype (η^2^ = 0.23), on the one hand, and hormonal treatment and dietary condition (η^2^ = 0.36), on the other hand, indicating an opposed effect of estradiol on saturate contents depending on the diet, whose magnitude depends on the genotype (Figure 2J). Such a combination of factors has a compensatory effect on brain cortex saturates, such that their contents remain stable between groups (Figure 2K), thus preserving their structural role in nerve cell membranes.

To our knowledge, this is the first demonstration that the effects of 17β-estradiol (mainly affecting plasmalogens and saturates) depend on the adequate provision of n-3 PUFAs, and on the presence of mutated AD proteins. This reinforces previous data demonstrating that estrogens may regulate brain lipid metabolism and homeostasis [57], yet with the influence of dietary conditions. Nevertheless, these estrogen effects will undoubtedly be modified throughout lifespan, adding another dimension of complexity, in particular during menopause and senescence [58,59].

### 3.1. Factor Effects on the Expression of Genes Involved in LCPUFA Biosynthesis in the Frontal Cortex

We have analyzed the expression of different genes encoding for LCPUFA biosynthesis and metabolism. These include *Elovl2-5* (*El*ongases *o*f *v*ery-*l*ong-chain fatty acids, and encoding for elongases 2, 4 and 5, respectively), *Fads1-2* (encoding for Δ5 and Δ6 desaturases) and *Acox1* (encoding for peroxisomal acyl-CoA oxidase 1, which catalyzes the chain shortening of 24-carbon intermediates by β-oxidation in peroxisomes, yielding C22:5n-6 and 22:6n-3 [60].

The results in Table 1 summarize the comparative expression of all genes in the frontal cortex of WT and APP/PS1 mice under dietary and hormonal treatments. The outcomes reveal a complex scenario in which gene expression is differentially affected (and to different extents), by the combination of the three factors. Comparison of DHA-Free and DHA-Suppl diets revealed no differences in WT animals despite quantitative and qualitative differences between diets (Table 1, upper row). This observation agrees with the reports in murine models showing that, contrary to liver, brain expression of LCPUFA biosynthetic genes remained unaltered by dietary n-3 PUFA restriction [27,35,44]. Conversely, significant changes were observed in the FAD genotype, both in placebo and in estrogen treatments (Table 1, upper row), consisting in the general downregulation of *Elovl4*, *Elovl5, Scd2* and *Fads1* genes in the DHA-Suppl diet as compared to the DHA-Suppl group. Such genotype-associated downregulation in response to dietary treatment in FAD animals was unexpected and suggests that APP/PSEN1 transgenes modify the expression pattern of LCPUFA biosynthesis machinery in the brain cortex. This is in contrast to the scenario in WT animals, where these genes are expressed rather constitutively at low expression rates (as compared to liver) insensitive to changes in dietary n-3 PUFA or DHA availability [35]. On the other hand, expression data in Table 1 (middle row) demonstrates the upregulation of *Acox1*, *Elovl2*, *Elovl4*, *Elovl5*, *Scd1* and *Scd2* genes in FAD animals, but not in WT littermates, exposed to the DHA-Free diet, irrespective of the hormonal treatment. These changes in FAD animals are physiologically relevant since levels of DHA and monounsaturated plasmalogens are nominally lower in DHA-Free diets, and these expression changes would provide a local mechanism to increase their contents in the brain cortex. In consonance, expression levels of the same genes under DHA-Suppl diets revealed their downregulation in FAD animals, but this effect was hormone-related and only observed in placebo-treated animals. At present, we have no reasonable hypothesis to explain the mechanism(s) underlying such genotype-related transcriptional regulation, but it is clearly associated to the presence of APP/PSEN1 transgenes.

Finally, we performed comparisons for the effects of hormonal treatment (Table 1, lower row) on gene expression. The effect of 17β-estradiol is evident in the DHA-Suppl diet, and exclusively in the APP genotype, where the presence of the hormone brings about the upregulation of all genes encoding for elongases, desaturase 5 and peroxisomal acyl-CoA oxidase 1. Thus, it seems that the effect of E2 is modulated by the type of diet and also by the presence of the APP genotype.

Overall, these results indicate that the genotype is the main factor in determining the rate of transcriptional regulation in frontal cortex, but the final scenario depends, to different extents, on the diet and the presence or absence of 17β-estradiol.

### 3.2. Frontal Cortex Lipid Classes Are Differentially Affected by the Presence of FAD Genotype, Circulating Estrogens, and the Dietary Condition

**Figure 3:**
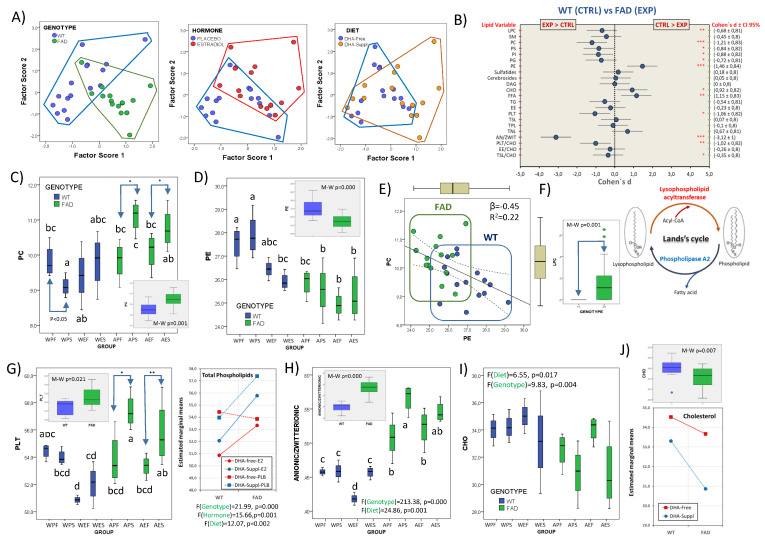
*Multifactor analyses of frontal cortex lipid classes*. (**A**) Scatterplots of factor scores from PC1 and PC2 with identification of main factor categories (genotype: WT and FAD; hormonal treatment: placebo and estradiol; dietary condition: DHA-Free and DHA-Suppl). (**B**) Forest plot for effect sizes (Cohen’s d) in lipid classes and related indexes between WT and FAD genotypes. Cohen’s d values are indicated along with their 95% confidence interval. Lipid variables were also submitted to Mann–Whitney U tests. *, **, and *** indicate *p*-values below 0.05, 0.01, and 0.005, respectively. (**C**) Boxplots for phosphatidylcholine (PC) contents in the eight experimental groups. * *p* < 0.05 between bars indicated by the arrows. In the inset, comparison of WT and FAD pooled subgroups. M-W: Mann–Whitney U test. (**D**) Boxplots for phosphatidylethanolamine (PE) contents in the eight experimental groups. * *p* < 0.05 between bars indicated by the arrows. In the inset, comparison of WT and FAD pooled subgroups. M-W: Mann–Whitney U test. (**E**) Regression plot for PC and PE contents in FAD and WT animals. Standardized regression coefficient (β) and determination coefficient (R^2^) of regression analyses for the whole dataset are indicated in the plot. (**F**) Boxplot representation for lysophosphatidylcholine (LPC) contents in WT and FAD groups. Sketch drawing of Land’s cycle showing lysophospholid formation. (**G**) Boxplots for total phospholipid (PLT) contents in the eight experimental groups. *, ** indicate *p* < 0.05 and *p* < 0.01 between bars indicated by the arrows. In the inset, comparison of WT and FAD pooled subgroups. M-W: Mann–Whitney U test. In the right graph, estimated marginal means for total phospholipids (PLTs) as a function of mouse genotype and hormonal treatment. F- and *p*-values for genotype, hormonal treatment and diet as main factors are indicated at the bottom of the graph. (**H**) Boxplots for anionic-to-zwitterionic ratio in the eight experimental groups. F- and *p*-values for genotype and diet as main factors are indicated at the bottom of the graph. In the inset, comparison of WT and FAD pooled subgroups. M-W: Mann–Whitney U test. (**I**) Effects of genotype and diet in the distribution of cholesterol (CHO) contents. Box plots for CHO contents in the eight experimental groups. F- and *p*-values for genotype and diet as main factors are indicated at the top of the graph. (**J**) Upper graph: comparison of WT and FAD pooled subgroups. M-W: Mann–Whitney U test. Lower graph: estimated marginal means for CHO as a function of mouse genotype and dietary treatment. In (**C**,**D**,**G**–**I**), different letters in bars within the same plot indicate statistical differences with *p* < 0.05 (Tuckey’s post hoc test). Experimental groups: APF: APP/PS1-Placebo-DHA-Free; APS: APP/PS1-Placebo-DHA-Suppl; AEF: APP/PS1-Estradiol-DHA-Free; AES: APP/PS1-Estradiol-DHA-Suppl; WPF: Wild-type-Placebo-DHA-Free; WPS: Wild-type-Placebo-DHA-Suppl; WEF: Wild-type-Estradiol-DHA-Free; WES: Wild-type-Estradiol-DHA-Suppl.

Results shown in Figure 1 showed that, besides fatty acids, lipid classes were also unevenly distributed over PC1, PC2 and PC3. Detailed examination of PC1 and PC2 (42.7% cumulative overall variance) indicated that both phospholipids and cholesterol exhibited highest extraction coefficients. Thus, in PC1, glycerophospholipids (PC, PS and PG) and cholesterol were oppositely related, while in PC2, PE and PI displayed opposed representation. Two-way ANOVA showed that the main effect was the genotype (F_1,40_ = 38.32, *p* = 0.000), followed by hormonal treatment (F_1,40_ = 14.13, *p* = 0.001) and lowest influence of diet (F_1,40_ = 9.64, *p* = 0.008) on PC1, without significant interactions between either factor (Figure 3A). Only for PC2 was a significant interaction observed for the genotype and hormonal status (F_1,40_ = 18.31, *p* = 0.000), which, in turn, were also very significant as main factors (hormonal treatment F_1,40_ = 13.23, *p* = 0.001; genotype F_1,40_ = 12.09, *p* = 0.002). Noticeably, the dietary contribution to factor scores was the lowest (in PC1) or null (in PC2). However, a certain influence of dietary condition for factor score 2 could be detected at the level of interaction between the three factors (F_1,40_ = 4.41, *p* = 0.046). These results indicate the selective modulation of frontal cortex lipid classes by the FAD genotype and not by dietary conditions, which dramatically differ from results of frontal cortex fatty acids. Such radical differences may be explained in terms of distinct biosynthetic scenarios, i.e., most LCPUFAs in brain tissue derive from liver biosynthesis or intestinal absorption (in the case of DHA-supplemented diets), and routed to the brain across the BBB, while lipid classes, particularly glycerophospholipids, cholesterol, and sphingolipids, are synthetized locally in nerve cells.

Analyses of individual lipid classes and related indexes as a function of genotype are shown in the forest plot in Figure 3B. The results indicate that PC levels were significantly higher and PE lower in APP than in WT genotypes, both displaying strong size effects. Similarly, PS and PI contents were higher in APP groups. These changes are reflected in the strong effect sizes (Cohen’s d = −1.06) for total phospholipid contents in APP groups (Figure 3B). Noticeably, the zwitterionic phospholipids PC and PE, accounting for >65% of total phospholipids, were differentially affected by the genotype (Figure 3C,D), PC being increased in FAD mice and PE decreased, as compared to WT. These changes were mutually related, with PC and PE negatively associated (R^2^ = 0.216, *p* = 0.007, β = −0.465) in the whole dataset (Figure 3E). Phospholipids (glycerophospholipids) are formed de novo in the Kennedy pathway using acyl-CoAs as donors. Subsequently, in Land’s cycle, cycles of deacylation (catalyzed by phospholipase A2s, PLA2) and reacylation (catalyzed by lysophospholipid acyltransferases, LPLATs) cause phospholipid remodeling, generating mature membranes with compositional and structural asymmetries [61,62,63]. As PC may be generated from PE in the last stage of Kennedy’s pathway, by the enzyme phosphatidylethanolamine N-methyltransferase (PEMT), we may conclude that the increased PC contents observed in FAD animals occur at the expense of the reduction in PE, as revealed by the negative relationship between PC and PE, as shown in Figure 3E. In support of this, levels of LysoPC, which derive from increased PC levels by the activity of phospholipase A2 by virtue of Land’s cycle, were consistently detected in FAD animals but totally absent in WT mice (Figure 3F). These results also agree with the observation that AA levels were higher in FAD animals receiving the DHA-Free diet (Figure 2F), presumably as a result of the same reaction catalyzed by phospholipase A2.

The evidence that the FAD genotype affects phospholipid remodeling is further revealed by the alteration of total phospholipids and the proportions of anionic-to-zwitterionic glycerophospholipids observed in Figure 3G and Figure 3H, respectively. These results indicate that the FAD genotype alters the physicochemical properties of frontal cortex membranes in two essential aspects: first, by changing the proportion of phospholipids to other bilayer constituents, i.e., cholesterol and sphingolipids (Figure 3B), involved in membrane physicochemical properties (see below); second, by increasing the proportion of anionic phospholipids, namely phosphatidylserine (PS), phosphatidylinositol (PI), and phosphatidylglycerol (PG), all exhibiting strong effect sizes (Figure 3B). In fact, the ratio to zwitterionic (PE + PC) phospholipids affects membrane geometry and curvature, and eventually membrane recycling, asymmetry and trafficking, protein sorting, crowding and insertion, and vesicle dynamics, amongst other features [64,65,66]. In the context of FAD, accumulating evidence suggests that lipid membranes and membrane curvature play key roles in amyloid protein aggregation, although the intermolecular forces that drive the interactions between Aβ-(1-40) or Aβ-(1-42) and the membranes vary in different membrane systems, membrane compositions, and physical properties [67]. For instance, a high positive curvature of lipid vesicles enhanced the association of Aβ with anionic phosphatidylglycerol membranes in the liquid-crystalline phase and with zwitterionic phosphatidylcholine membranes in the gel phase [68]. A much more complex situation occurs in nerve cell membranes, where lipid changes associated to the FAD genotype drive a global effect on phospholipid groups and remodeling, as shown here. Thus, an essential connection may be surmised between phospholipid remodeling, leading to a high anionic-to-zwitterionic proportion, and amyloid oligomerization and burden, as we have and others have reported in this same FAD model [21,67].

Besides the genotype, we demonstrate that membrane phospholipids are also modulated by the hormonal and dietary treatments (Figure 3G, right panel). Indeed, MANOVA (multiple analyses of variance) revealed strong effects for hormone (F = 27.26, Wilks’ λ = 0.104, η^2^ = 0.896) and its interaction with genotype (F = 26.02, Wilks’ λ = 0.109, η^2^ = 0.891). Of particular interest is PS, the most abundant anionic phospholipid, which exhibits a strong dependence on the presence of estradiol (F_1,40_ = 31.14, *p* = 0.000, η^2^ = 0.565), the interaction of the hormonal status with the genotype (F_1,40_ = 5.63, *p* = 0.026, η^2^ = 0.190), and the three factors (F_1,40_ = 7.05, *p* = 0.014, η^2^ = 0.227). These effects will be explored in detail in the next paragraph.

### 3.3. Involvement of Phospholipid Remodeling

**Figure 4:**
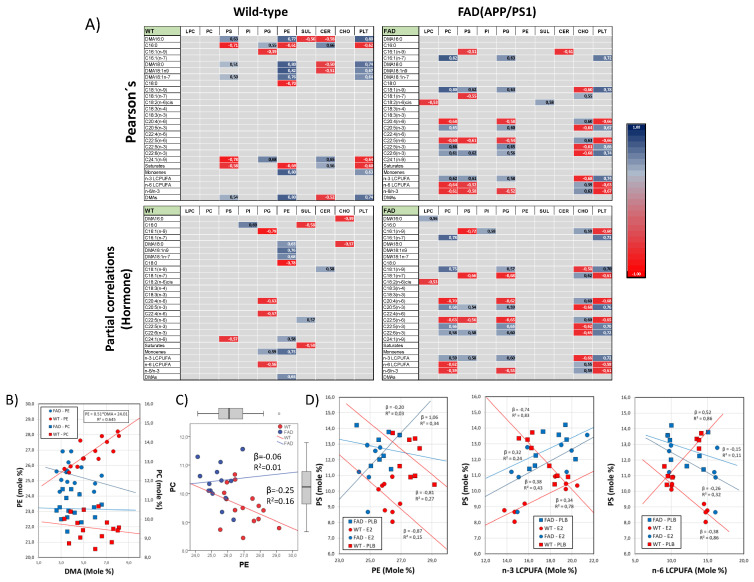
*Empirical evidence for phospholipid remodeling in the frontal cortex*. (**A**) Analyses of Pearson’s correlation (upper matrixes) and partial correlation controlling for hormonal condition (lower matrixes) for lipid classes and fatty acids in WT and FAD animals. Only significant correlation values are indicated in the tables, and they are colored according to the scale on the right. (**B**) Regression analyses for the relationships between PE or PC and total DMA contents. Regression equation and R^2^ are shown for only the significant linear association found (PE vs. DMAs in WT animals). (**C**) Analyses of bivariate association between PC and PE in WT and FAD animals. (**D**) Regression analyses for PS as a dependent variable and PE (left), n-3 LCPUFAs (middle) and n-6 LCPUFAs (right) as independent variables. Regression (β) and determination coefficients (R^2^) are indicated for each regression line in the plots.

We next explored the potential effects of genotype and hormonal treatment on the relationships between lipid classes and fatty acids. We performed Pearson’s correlation analyses on their bivariate relationships. The results are shown in Figure 4A. At first glance, the heatmaps in the upper panel reveal a clear differentiation between WT and FAD animals, affecting several bivariate associations. Of particular interest are the differences in the correlation coefficients for PC, PS, PE, or total phospholipids (PLTs) and the different fatty acids. The interpretation of these observations is that the presence of the FAD genotype alters the distribution of the different fatty acids between phospholipids, likely by altering the combinatorial esterification of phospholipid’s glycerol backbones by acyl chains. A striking example of these genotype-related alterations is illustrated in Figure 4B, where the strong positive association between DMAs and PE, corresponding to PE plasmalogens (the most abundant fraction of membrane plasmalogens), vanished in the cortexes of FAD animals. Such an effect was specific for PE plasmalogens and was not observed for the corresponding PC species (Figure 4B).

Bivariate relationships between phospholipid levels were also affected by the presence of the FAD genotype, in particular PE and PC, whose relative independence in the cortexes of WT animals becomes a strong negative association in FAD littermates (Figure 4C, left plot), supporting the hypothesis suggested above that the FAD genotype affects Kennedy’s and Land’s cycles.

Moreover, our analyses also revealed that the precise effect of genotype on particular lipid class–fatty acids pairs might be influenced by the hormonal status. Indeed, using partial correlation analyses, we demonstrate that some of the most robust associations disappeared when the analyses include the hormonal status factor as control variable (Figure 4A, lower matrixes). Comparatively, changes in partial correlations versus Pearson’s correlations are more evident in WT animals than in FAD animals, indicating lower sensitivity to hormonal influences in the FAD genotype.

The influence of hormonal status on genotype effects is neatly observed in the esterification of phosphatidylserine by n-3 and n-6 LCPUFAs (Figure 4D, middle and right plots, respectively). These fatty acids are bound to the *sn*-2 position of the PS glycerol backbone. Correlation analyses indicate that PS is oppositely related to n-3 and n-6 LCPUFAs in FAD animals but unrelated in WT counterparts (Figure 4A, Pearson’s correlation). However, such relationships disappeared when the hormonal condition was used as a covariate in the analyses (Figure 4A, partial correlations). Detailed regression analyses revealed that while n-3 LCPUFAs were positively correlated to PS in WT brains exposed to estradiol, their absence caused the opposite effect (Figure 4D, middle plot). The relationships with n-6 LCPUFAs mirrored those of n-3 LCPUFAs, i.e., the absence of estradiol reversed the negative association with PS (Figure 4D, right plot). Further, we observed that these changes in PS esterification were paralleled by the hormone-dependent association between PS and PE (Figure 4C), which were negatively related in WT brains but positively related or unrelated in FAD animals.

We may conclude that the genotype is a critical factor, not only in setting membrane phospholipid proportions but also their fatty acid compositions. The extent of such a phospholipid remodeling effect of estradiol is notably dependent on the genotype, FAD animals being much less sensitive to the presence of 17β-estradiol. To our knowledge, this is the first report showing a direct linkage between AD-linked mutations, brain estrogen, and phospholipid metabolism.

### 3.4. Structural and Biophysical Correlates of Changes in Lipid Profiles

**Figure 5:**
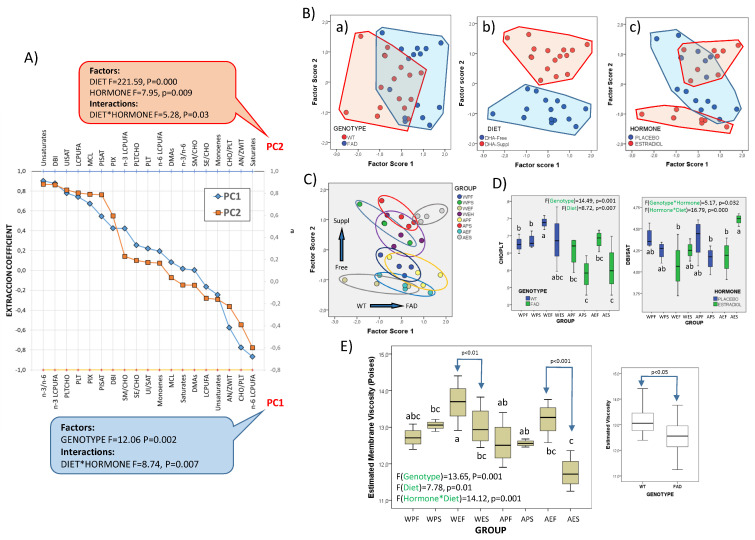
*Structural and biophysical correlates of frontal cortex lipid changes*. (**A**) Extraction coefficients from factor analyses of lipid-related parameters with relevance in membrane physicochemical properties. Factors in PC1 and PC2 were reordered according to their extraction coefficients. F- and *p*-values from the two-way ANOVA for main factors and their interactions are shown for each principal component. (**B**) Scatterplots of factor scores from PC1 and PC2 with identification of factor categories (genotype: WT and FAD; dietary condition: DHA-Free and DHA-Suppl; hormonal treatment: placebo and estradiol; (**C**) identification of experimental groups in the 2D plot of factor score. (**D**) Boxplots for the cholesterol/total phospholipid (CHO/PLT) ratios and double bond index/saturates (DBI/SAT) in the eight experimental groups. Bar colors refer to genotype (left plot) and hormonal condition (right plot). F- and *p*-values for main factors and interactions are indicated at the top of each graph. (**E**) Boxplots for estimated microviscosity values of nerve cell membranes in the experimental groups. Right plot: Comparative estimation of membrane viscosity in the brain cortex of WT and FAD animals. In (**D**,**E**), different letters in bars within the same plot indicate statistical differences with *p* < 0.05 (Tuckey’s post hoc test). Experimental groups: APF: APP/PS1-Placebo-DHA-Free; APS: APP/PS1-Placebo-DHA-Suppl; AEF: APP/PS1-Estradiol-DHA-Free; AES: APP/PS1-Estradiol-DHA-Suppl; WPF: Wild-type-Placebo-DHA-Free; WPS: Wild-type-Placebo-DHA-Suppl; WEF: Wild-type-Estradiol-DHA-Free; WES: Wild-type-Estradiol-DHA-Suppl.

The potential impact of lipid changes on the structural and biophysical properties of nerve cell membranes was further assessed using multivariate analyses of physicochemical-related variables. The results are summarized in Figure 5. First, in Figure 5A, we observed a positive association between the total n-3/n-6 ratio, n-3 LCPUFAs, and phospholipids (PLTs) and PC1, and a negative association between these variables and n-6 LCPUFAs, cholesterol-to-phospholipids (CHO/PLT) and Anionic-to-Zwitterionic phospholipids (AN/ZWIT). PC2 was positively related to the double bond index (DBI), total LCPUFAs, mean carbon chain length (MCL), and DBI-to-saturates (UISAT) ratio, and negatively related to saturates. The analyses of factors and interactions revealed a strong effect of genotype (Figure 5(Ba)) and a significant interaction between the diet and hormonal status in PC1. A highly significant effect of diet was demonstrated for PC2 (Figure 5(Bb)) as well as a moderate effect of hormone treatment and hormone–diet interaction (Figure 5(Bc)).

Besides contributions of n-3 and n-6 LCPUFAs to PC1 (Figure 5A), which are strictly diet-related and determine the n6/n3 ratio, the other variables included in PC1 and PC2 are critical determinants of membrane physiological properties, i.e., membrane fluidity, domain organization, membrane packing, and curvature, amongst other features [65,69,70,71,72].

Estimation of membrane viscosity (in reciprocity to fluidity) from lipid profiles was performed following the statistical approaches reported for mouse brains [70,72]. A predictive multivariable function based on multiple regression analyses, which integrates the contributions of the phospholipid-to-cholesterol ratio, unsaturation-index-to-saturates ratio, average carbon chain length, and anionic-to-zwitterionic-phospholipids ratio were found to accurately predict membrane microviscosity (R^2^ = 0.68, *p* < 0.005) [73].

Therefore, we performed one-way ANOVA on these different predictors to detect differences between experimental groups (Figure 5C) and two-way ANOVA to assess the contributions of the three factors and their interactions. The results demonstrate that differences between groups for the CHO/PLT ratio are mainly determined by the genotype (η^2^ = 0.38), followed by dietary treatment (η^2^ = 0.27), while the inter-group differences in the essential component for membrane unsaturation (DBI/SAT) was the result of strong hormonal interactions with the genotype (η^2^ = 0.18), on the one hand, and diet (η^2^ = 0.41), on the other (Figure 5D).

Based on these parameters, we have estimated membrane microviscosity at the membrane plane (Figure 5E). The results indicate strong and significant differences between groups, with main effects attributable to the genotype (η^2^ = 0.472; Figure 3F, right panel) and hormonal condition (η^2^ = 0.424), as well as a strong interaction between the genotype and dietary condition (F = 11.75, *p* = 0.002, η^2^ = 0.329). The evidence that the hormonal condition determines the response to diet is illustrated in Figure 5E, where it is only in the presence of estradiol that significant differences between DHA-Free and DHA-Suppl are detected, both in WT and FAD genotypes.

The membrane viscosity values estimated here fall within the range of values reported for mouse brains and reflect the steady state for lipid constituents endowed with opposed effects on lipid bilayers, i.e., LCPUFAs vs. cholesterol, total saturated vs. unsaturated fatty acids, phospholipids vs. sterols (cholesterol and sterol esters), or zwitterionic to anionic or total phospholipids. Overall, our results reveal a profound effect of the FAD genotype on the physicochemical phenotype of frontal cortex nerve cells.

## 4. Conclusions

In summary, we demonstrate that the modulation of brain cortex lipids via dietary supply of n-3 PUFAs and the estrogen status are substantially altered by the presence of a familial-type Alzheimer’s disease genotype. Overall, our results reveal that the influence of these factors in the lipid matrix is not entirely independent but is rather marked by significant interactions between factors, which differentially affect brain lipid homeostasis. The results reveal alterations in the distribution and proportion of fatty acids (n-3 and n-6 LCPUFAs, saturates, and plasmalogens) but also in the expression of genes involved in LCPUFA biosynthesis, which vary depending on the dietary supply in a genotype-depending manner. Moreover, we demonstrate a global change in phospholipid levels and phospholipid remodeling, under the strong influence of estrogen and the FAD genotype. We finally showed that such alterations in fatty acids and phospholipids due to the FAD genotype have a great impact on nerve cell membrane properties.

Though the findings described in this study may be relevant from a neuropathological perspective, their relevance from a translational point of view and their clinical implementation to humans requires extensive investigation.

## Figures and Tables

**Table 1:** Gene expression in the frontal cortex.

**WT**				**WT**				**FAD**				**FAD**		
**PLACEBO**				**E2**				**PLACEBO**				**E2**		
**DHA-Free vs. DHA-Suppl**				**DHA-Free vs. DHA-Suppl**				**DHA-Free vs. DHA-Suppl**				**DHA-Free vs. DHA-Suppl**		
**Gene**	**Expression**	***p*-Value**		**Gene**	**Expression**	***p*-Value**		**Gene**	**Expression**	***p*-Value**		**Gene**	**Expression**	***p*-Value**
*Elovl2*	0.974	0.833		*Elovl2*	0.953	0.769		*Elovl2*	0.277	0.067		*Elovl2*	0.661	0.067
*Elovl4*	0.988	0.887		*Elovl4*	0.935	0.61		*Elovl4*	0.273	0.056		*Elovl4*	0.66	0.032
*Elovl5*	0.803	0.698		*Elovl5*	1.438	0.571		*Elovl5*	0.054	0.063		*Elovl5*	0.707	0.042
*Scd1*	1.521	0.079		*Scd1*	1.074	0.536		*Scd1*	0.554	0.067		*Scd1*	0.741	0.137
*Scd2*	1.143	0.131		*Scd2*	1.116	0.571		*Scd2*	0.334	0.029		*Scd2*	0.749	0.013
*Fads1*	0.782	0.388		*Fads1*	1.479	0.343		*Fads1*	0.057	0.029		*Fads1*	0.731	0.053
*Fads2*	0.959	0.516		*Fads2*	1.123	0.287		*Fads2*	0.923	0.759		*Fads2*	0.903	0.426
**PLACEBO**				**PLACEBO**				**E2**				**E2**		
**DHA-Free**				**DHA-Suppl**				**DHA-Free**				**DHA-Suppl**		
**WT vs. FAD**				**WT vs. FAD**				**WT vs. FAD**				**WT vs. FAD**		
**Gene**	**Expression**	***p*-Value**		**Gene**	**Expression**	***p*-Value**		**Gene**	**Expression**	***p*-Value**		**Gene**	**Expression**	***P*-Value**
*Acox1*	1.416	0.033		*Acox1*	0.184	0.034		*Acox1*	1.473	0.137		*Acox1*	1.07	0.443
*Elovl2*	1.917	0.036		*Elovl2*	0.542	0.173		*Elovl2*	1.826	0.026		*Elovl2*	1.266	0.275
*Elovl4*	1.467	0.029		*Elovl4*	0.397	0.012		*Elovl4*	1.596	0.032		*Elovl4*	1.117	0.138
*Elovl5*	1.607	0.053		*Elovl5*	0.105	0.078		*Elovl5*	2.113	0.238		*Elovl5*	1.038	0.741
*Scd1*	1.986	0.068		*Scd1*	0.723	0.285		*Scd1*	1.6	0.000		*Scd1*	1.102	0.433
*Scd2*	1.635	0.053		*Scd2*	0.478	0.096		*Scd2*	1.599	0.054		*Scd2*	1.085	0.465
*Fads1*	1.069	0.540		*Fads1*	0.079	0.054		*Fads1*	1.434	0.425		*Fads1*	0.708	0.024
*Fads2*	1.211	0.154		*Fads2*	1.162	0.509		*Fads2*	1.299	0.188		*Fads2*	1.041	0.684
**WT**				**WT**				**FAD**				**FAD**		
**DHA-Free**				**DHA-Suppl**				**DHA-Free**				**DHA-Suppl**		
**PLACEBO vs. E2**				**PLACEBO vs. E2**				**PLACEBO vs. E2**				**PLACEBO vs. E2**		
**Gene**	**Expression**	***p*-Value**		**Gene**	**Expression**	***p*-Value**		**Gene**	**Expression**	***p*-Value**		**Gene**	**Expression**	***p*-Value**
*Acox1*	0.958	0.898		*Acox1*	1.091	0.801		*Acox1*	1.004	1.000		*Acox1*	6.20	0.088
*Elovl2*	1.107	0.884		*Elovl2*	1.086	0.841		*Elovl2*	1.042	0.755		*Elovl2*	2.51	0.061
*Elovl4*	0.975	0.793		*Elovl4*	0.927	0.593		*Elovl4*	0.992	0.967		*Elovl4*	2.51	0.081
*Elovl5*	0.788	0.741		*Elovl5*	1.412	0.809		*Elovl5*	1.044	0.735		*Elovl5*	13.98	0.082
*Scd1*	1.231	0.299		*Scd1*	0.868	0.254		*Scd1*	0.991	0.967		*Scd1*	1.32	0.384
*Scd2*	1.032	0.798		*Scd2*	1.01	0.869		*Scd2*	1.012	0.934		*Scd2*	2.32	0.09
*Fads1*	0.794	0.586		*Fads1*	1.499	0.022		*Fads1*	1.073	0.447		*Fads1*	13.45	0.061
*Fads2*	0.946	0.459		*Fads2*	1.107	0.214		*Fads2*	1.01	0.846		*Fads2*	0.98	0.901

Expression is indicated as fold times. Pale blue: downregulation. Pink: upregulation.

## Data Availability

All data and analyses are available upon request.

## Supplementary Materials

The following supporting information can be downloaded at: https://www.mdpi.com/article/10.3390/genes15060810/s1, Table S1: Fatty acid composition (g/kg) of experimental diets.
