# Supplementary material for: Multifactor Analyses of Frontal Cortex Lipids in the APP/PS1 Model of Familial Alzheimer’s Disease Reveal Anomalies in Responses to Dietary n-3 PUFA and Estrogenic Treatments
Source: Genes (Basel). 2024 Jun 19;15(6):810. doi: 10.3390/genes15060810 (PMC11202691; doi:10.3390/genes15060810)
Supplement: Supplementary file 1 [file genes-15-00810-s001.zip › genes-3012751-supplementary.pdf]

Multifactor analyses of frontal cortex lipids in the APP/PS1 model of familial Alzheimer´s Disease disclose anomalies in the responses to dietary n-3 PUFA and estrogenic treatments

Mario Díaz

Membrane Physiology and Biophysics. Department of Physics. School of Sciences, University of La Laguna, 38206 Tenerife, Spain, and Instituto Universitario de Neurociencias (IUNE), University of La Laguna, 38206 Tenerife. Spain.

madiaz@ull.es

madiaz@ull.edu.es

Supplementary material

Supplementary table S1. Fatty acid composition (g/kg) of experimental diets.

	DHA-Suppl	DHA-Free
14:0	2.34	0.32
16:0	9.31	7.42
16:1(n-9)	0.24	0.06
16:2(n-4)	0.16	0.00
18:0	2.54	2.49
**18:1(n-9)**	**8.91**	**19.32**
**18:2(n-6)**	**25.20**	**51.44**
**18:3(n-3)**	**0.58**	**0.19**
18:4(n-3)	1.11	0.00
20:0	0.33	0.27
20:1(n-9)	1.54	0.14
20:2(n-6)	0.10	0.00
**20:4(n-6)**	**0.30**	**0.00**
20:4(n-3)	0.35	0.00
**20:5(n-3)**	**3.84**	**0.00**
22:0	0.11	0.15
22:1(n-9)	0.22	0.01
21:5(n-3)	0.13	0.00
22:5(n-6)	0.13	0.00
22:5(n-3)	0.67	0.00
24:0	0.00	0.09
**22:6(n-3)**	**6.31**	**0.00**
24:1(n-9)	0.30	0.12
